# Improved Deep Convolutional Neural Network to Classify Osteoarthritis from Anterior Cruciate Ligament Tear Using Magnetic Resonance Imaging

**DOI:** 10.3390/jpm11111163

**Published:** 2021-11-09

**Authors:** Mazhar Javed Awan, Mohd Shafry Mohd Rahim, Naomie Salim, Amjad Rehman, Haitham Nobanee, Hassan Shabir

**Affiliations:** 1 School of Computing, Faculty of Engineering, Universiti Teknologi Malaysia, Skudai 81310, Malaysia; shafry@utm.my (M.S.M.R.); naomie@utm.my (N.S.); 2 Department of Software Engineering, University of Management and Technology, Lahore 54770, Pakistan; hassangillani@outlook.com; 3 Artificial Intelligence and Data Analytics Research Laboratory, CCIS, Prince Sultan University, Riyadh 11586, Saudi Arabia; rkamjad@gmail.com; 4 College of Business, Abu Dhabi University, P.O. Box 59911, Abu Dhabi 59911, United Arab Emirates; 5 Oxford Centre for Islamic Studies, University of Oxford, Oxford OX1 2J, UK; 6 School of Histories, Languages and Cultures, The University of Liverpool, Liverpool L69 3BX, UK

**Keywords:** anterior cruciate ligament, osteoarthritis, deep learning, classification, public health, healthcare, diagnosis, convolutional neural network, knee bone, radiographic image analysis, human and health

## Abstract

Anterior cruciate ligament (ACL) tear is caused by partially or completely torn ACL ligament in the knee, especially in sportsmen. There is a need to classify the ACL tear before it fully ruptures to avoid osteoarthritis. This research aims to identify ACL tears automatically and efficiently with a deep learning approach. A dataset was gathered, consisting of 917 knee magnetic resonance images (MRI) from Clinical Hospital Centre Rijeka, Croatia. The dataset we used consists of three classes: non-injured, partial tears, and fully ruptured knee MRI. The study compares and evaluates two variants of convolutional neural networks (CNN). We first tested the standard CNN model of five layers and then a customized CNN model of eleven layers. Eight different hyper-parameters were adjusted and tested on both variants. Our customized CNN model showed good results after a 25% random split using RMSprop and a learning rate of 0.001. The average evaluations are measured by accuracy, precision, sensitivity, specificity, and F1-score in the case of the standard CNN using the Adam optimizer with a learning rate of 0.001, i.e., 96.3%, 95%, 96%, 96.9%, and 95.6%, respectively. In the case of the customized CNN model, using the same evaluation measures, the model performed at 98.6%, 98%, 98%, 98.5%, and 98%, respectively, using an RMSprop optimizer with a learning rate of 0.001. Moreover, we also present our results on the receiver operating curve and area under the curve (ROC AUC). The customized CNN model with the Adam optimizer and a learning rate of 0.001 achieved 0.99 over three classes was highest among all. The model showed good results overall, and in the future, we can improve it to apply other CNN architectures to detect and segment other ligament parts like meniscus and cartilages.

## 1. Introduction

The knee is the strongest joint in the human body. It is secured by ligament structures protect the knee joint’s bone elements [1,2]. Every year, there are about 25,000 people with ACL ruptures [3]. The ACL is one of the most commonly injured ligaments in the knee. The ACL crosses inside the knee connecting the thigh bone to the leg. The lesion mechanisms causing ACL tears are lateral rotation, backward displacement, or sideways impact on the knee, while the ligaments are non-elastic solid fibers that connect our bones [4,5]. ACL tears can cause knee pain, swelling, instability, osteoporosis, and osteoarthritis [6].

Knee osteoarthritis (KOA) is degenerative, severe, and painful, develops slowly over time, and affects a large population worldwide in all age groups; knee osteoarthritis is caused by a breakdown of cartilage and ruptured in the anterior cruciate ligament [7,8]. However, it is difficult for the radiologist to detect different wounds from radiological scans, and scans can be time consuming and error prone. There are various methods to identify osteoarthritis in an ACL tear of the knee by looking at loads from the gait, biochemical changes, and radiology images like X-rays, CT scans, and magnetic resonance imaging (MRI) [9].

MRI uses very strong magnetic radio waves and a computer to take pictures of the inside of the body. MRI is better to identify injuries inside the body, such as torn anterior cruciate ligament. MRI is a 3D picture that slices through the knee in three planes: sagittal, coronal, and axial [10]. The varying grades of ACL tears can be better identified through MRI [11,12]. The easiest way to find the ligaments is on the sagittal, which is the side view of the MR slice [13,14].

Deep learning is a machine learning branch that automatically identifies features from images [15,16,17,18]. Convolutional neural network (CNN) models are good at classifying microscopic images through deep learning [19,20]. Recently, applied machine learning and deep learning models have been applied to various diseases, such as COVID-19 [21,22], acute leukemia [23,24], schistosomiasis [25], lung diseases [26,27], diabetic retinopathy [28], dental surgery [29], retinal diseases [30], thyroid surgery [31], drug diagnosis [32], brain tumors [33,34,35], and health diseases [36]. Accurate automatic image classification, segmentation, and detection is a challenge in computer vision, particularly in medical research. Image segmentation is clinically important to determine the bone tissues and classify the segmentation result through MRI [37,38]. Segmentation of connected components through labeling scans with region-based levels set was performed good results for MRI [39,40].

Various previous studies were implemented applying deep learning models to MR images to classify ACL injury. Our dataset was obtained from the researchers Stajduhar et al. [41]. They applied a semi-automated approach in which histogram-oriented features were extracted manually and automatically classified two classes of ACL tears from knee MRIs by support vector machine. The model running time to diagnose was within one second. The area under the curve (AUC) of partial and complete tears was found to be only 0.894 and 0.943, respectively, using 10-fold cross-validation. The limitation of the study was the low performance of the model due to a lack of distinction between images showing partial injuries and non-injured knees. Secondly, the study considered only two classes, partial tear and completely ruptured tears. Bien et al. [42] extracted features from a CNN with a pre-trained AlexNet transfer learning model with three logistic regression functions for abnormalities, ACL tear, and meniscus, which they trained on MRNet. The experimental results were obtained from the validation set of same knee MRI data [41] with an AUC, specificity, sensitivity, and accuracy of 0.911, 0.968, 0.759, and 0.867, respectively. The limitation of the study was a lack of surgical confirmation of the validation dataset. Tsai et al. [43] also trained a CNN architecture called efficiently-layered network (ELNet) on MRNet and validated on MRIs of knee ACL tears. The model was light weighted and contained approximately 0.2 million parameters only. However, the limitation in terms of accuracy was that 90% was not a good performance for ACL tears evaluated on MRNet, and the study only reported AUC in case of the knee MRI dataset. Liu et al. [44] performed a classification task of 175 ACL tears. MRIs were evaluated through a densely connected convolutional network (DenseNet). The diagnostic performance of VGG16, AlexNet, and the proposed DenseNet of 161 layers in detecting ACL tears was evaluated by AUC as 0.950, 0.90, and 0.98, respectively. However, this study only considered three CNN models in a cascaded way, not as a single pipeline, which leads to a high burden on training. Furthermore, there was no verification of bias, and the dataset for training was significantly smaller. Namiri et al.’s [45] study of hierarchical severity used 1243 knee MRIs with four classes of ACL tears. The CNN model was tested on 2D as well as 3D CNN models. The overall performance of the 2D CNN was higher than that of the 3D CNN, but without transfer learning it was worse. The limitation of the study was that subcategories of partial tears were not classified due to the limited size of the test set. The MRI grades were dependent on radiologists. Kapoor et al. [46] compared different deep learning models, i.e., CNN, deep convolutional network (DCN), and recurrent neural network (RNN), as well as machine learning algorithms, i.e., logistic regression and SVM. These models were applied to a knee MRI dataset. Although the study was applied extensive models, their performance was lower in the case of SVM, CNN, DCN, and RNN.

Awan et al.’s [47] state-of-the-art work recently implemented a CNN architecture of a customized ResNet-14 trained on knee MRI datasets. The detection of ACL tears was performed at an average accuracy of 92% for three classes. The model was tested not only with random splitting but also by 3-fold and 5-fold cross-validation. The average accuracy was 92%, and AUC was reported to be 0.98 after hybrid class balancing and real-time augmentation with 5-fold cross-validation. The limitation of their model was that it took a lot of processing time to train even on a graphical processing unit (GPU).

In summary, several methods were proposed in the literature for the automated classification of ACL tears in MRI. These studies used varying numbers of images and datasets from multiple sources. Moreover, different approaches were used to evaluate the performance of the models that have drawbacks and are time-consuming even when automated. The quick diagnosis of various knee abnormalities is a challenging task due to the variability in MR images. This study proposes a convolutional neural network deep learning model that automatically classifies knee ACL tears from MR images. The modified convolutional neural network (CNN) performed at an accuracy of above 96% after hyperparameter tuning within few seconds.

Furthermore, our proposed model significantly predicted the ruptured tears of ACL to detect osteoarthritis. The contributions of our paper are to develop a modified CNN, after adjusting hyperparameters; to classify healthy, partially injured, and fully ruptured ACLs; and to extensively explore the experimental results by plotting and evaluating accuracy, precision, sensitivity, specificity, F1-score, ROC AUC, training, and test loss values. As per our knowledge, our proposed CNN network is more effective and efficient than the other studies reported previously. Therefore, the proposed model could be used for rapidly detecting ACL ruptures in sportspersons as well as osteoarthritis patients in hospitals.

## 2. Materials and Methods

This Section 2 shows the materials and methods used in this study. Section 2.1 describes the dataset. Section 2.2 explains the exclusion and labeling criteria. Section 2.3 describes the data pre-processing of the MR images used in the proposed methods. Finally, the proposed CNN model is presented in Section 2.4.

### 2.1. Data Collection Description

The Stajduhar et al. [41] collected 969 12-bit grayscale DICOM MRI volumes of the left or the right knee at the clinical hospital center in Rijeka, Croatia, between 2006 and 2014. The detailed protocols are summarized as follows:

Scanner manufacturer = Siemens Avanto, MR scanner proton density (PD) = 1.5 T weighted fat suppression, MR plane = DICOME sagittal volumes, plane spaces on X and Y axis = 0.56 mm, high resolution, plane spaces on Z-axis between slices = 3.6 mm, blur resolution, and slice thickness was 3 mm.

The following ten variables were included: exam Id, Serial No, Knee Left Right (KneeLR), Region of Interest X axis (roiX), Region of Interest Y-axis (roiY), Region of Interest Z-axis (roiZ), Region of Interest Height (roiHeight), Region of Interest Width (roiWidth), Region of Interest Depth (roiDepth), and ACL Diagnostics of three classes.

### 2.2. Data Exclusion and Labelling Criteria

The study’s authors manually inspected all volumes and categorized them into three degrees of ACL tears under the supervision of four expert radiologists with experience in musculoskeletal injuries. Some volumes were excluded from the study after evaluation based on the following reasons: (1) DICOM slices missing (three volumes); (2) abnormal characteristics in knees, severe osteoarthritis, or knee after ACL reconstruction (22 volumes); and (3) 27 cases were excluded where radiologists agreed on the diagnosis.

The University’s orthopedic clinic in Lovran confirmed 25 patients with fully ruptured ACL and 3 patients diagnosed with partial ACL ruptures. All patients were first diagnosed by a clinical exam performed by an orthopedist or traumatologist. The exam was performed because the patients demanded it due to pain and the knee “dropping” while walking or running. They had a positive anterior drawer test or Lachman test. Some patients had an older injury, whereas other injuries were more recent. The finding was confirmed only for those patients that underwent surgery.

**Figure 1:**
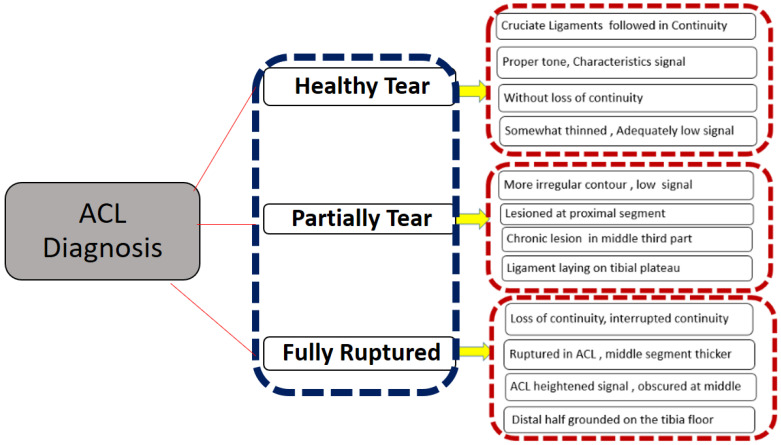
The ACL conditions of the extracted diagnosis criteria.

Thus, the collection of 917 knee datasets consisted of 690 healthy patients (75.2%), 172 partial ruptures (18.8%), and 55 fully ruptured (6.0%) ACLs. The detailed description of the inclusion of three ACL tear diagnosis conditions as shown in Figure 1.

Furthermore, this study was approved by the Ethics Committee of CHC Rijeka on 30 August 2014. Moreover, on 23 May 2017, it received the Ethics Committee’s approval to make the data publicly available. The data we used was anonymized. Therefore, we were unable to share more detailed MR sequence parameters or patient characteristics.

### 2.3. Data Pre-Processing

Each image in the dataset was of the size 330 × 330 × 32 (width, height, and depth, respectively), where depth is the number of slices in the images. The image size is too big to handle at a low cost, and our region of interest is also a small part of the image. Therefore, we decided to take only the region of interest (ROI) into account. After ROI extraction, the new shape of all images was (90 × 90 × 1).

### 2.4. Convolutional Neural Network Methodology

The traditional convolutional neural network is a feed-forward neural network. A CNN, also known as “ConvNet”, has five layers. A CNN is identical to ordinary neural networks, such as a multilayer perceptron. The CNN model extracts features from the corners and edges and other high-level features in the first layers [48].

We propose two different variants of a convolutional neural network to perform deep learning on our knee MR images. Firstly, we trained a classical LeNet-5 [49] CNN architecture, which we refer to as the standard CNN of five layers without input and output layers but with different parameters. Secondly, we enhanced the layers in the CNN model, referred to as the customized CNN.

Detailed descriptions of both of our variants of layers, filters, stride, and activation functions are given below:

1. Convolutional Layer

The image (90 × 90) is fed into the convolutional layer. The number of filters that are applied across the input at a 2 × 2 stride is 20. The depth of the filter is the same as the input.

The convolution operation involves the element-wise product of 20 filters in the image and then summing those values for every sliding action. The process of applying a filter masks the image in the form of matrices and gets features from images. Equation (1) shows the function of the convolutional kernel where a feature map is calculated for the input image of kernel *m*_1_. The output is G, with the *x*_th_ feature map of layer *l*, and Bias and filter (*F* (*l*)) are matrices connecting the *y*_th_ feature map.

(1) $$G_{x}^{\left(l\right)}=\overset{m_{1}\left(l-1\right)}{\underset{y=1}{\sum }}F_{x,y}^{\left(l\right)}*\;G_{y}^{\left(l-1\right)}+Bias{\;}^{\left(l\right)}$$

2. Activation Layer

The non-linear activate function rectified linear unit (ReLu) is used between subsequent convolutional layers [50]. The non-linearity function is explained in the Equation (2), where the layer l is a non-linearity layer. $G_{x}^{l}$ is generated through the feature volume $G_{x}^{\left(l-1\right)}$ from a previous layer_(*l*−1)_.

(2) $$G_{x}^{l}=f\left(G_{x}^{\left(l-1\right)}\right)\;$$

The ReLu function sets negative values to zero. Equation (3) is a function of maximum value. The objective of the activation function ReLu is to get the output from the neural network, and the final hidden layers are processed to get information that resides in the images which can further be evaluated on unseen images to get the prediction of classes.

(3) $$G_{x}^{l}=\max\left(0,\;G_{x}^{\left(l-1\right)}\right)$$

3. Pooling Layer

A pooling layer has the function of downsampling features. Max-pooling compresses the image and enhances the features. The filter returns the maximum value among the features. The sliding window, which skips along the width and height, is used at a stride of 2 × 2.

(4) $$\left[\frac{n+2P-f}{2}+1\right]$$

4. Fully Connected Layer

The above three layers extract features from knee MR images. Then they are passed into a fully connected layer called a dense layer. In this layer, every input is connected to every output by weights. It serves the purpose of doing actual classification.

The input is flattened before it is fed into the dense layer.

5. Output Layer

The output dense layer classifies the image into three neurons. These three classes are healthy, partially ruptured, and fully ruptured. The softmax activation function is applied in this layer.

#### 2.4.1. Standard CNN Modified Architecture

**Figure 2:**
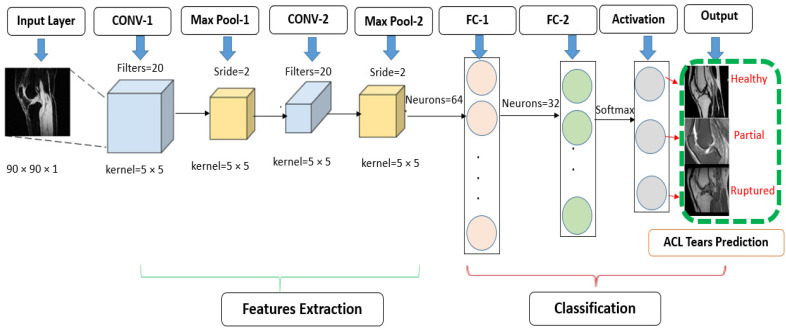
Modified standard 5 layer CNN architecture for the prediction of ACL tears.

From the input layer, knee images are passed through subsequent layers to classify tears as healthy, injured, and fully ruptured ACLs. Our modified standard CNN model variant was inspired by the LeNet-5 CNN architecture of five layers but with different parameter settings. The standard model had two convolutional layers fixed with 20 filters, a kernel size of 5 × 5, and a stride of 2 × 2. Through maximum pooling instead of average pooling, we modified this to a filter size of 2 × 2 and a stride of 2 × 2. We also changed activation function between the layers from tanh to ReLu. Figure 2 illustrates the standard model with two features extraction layers. The classification section is our output prediction. After feature extraction, we used two fully connected layers with 64 and 32 neurons, respectively. The last layer is our softmax activation layer of three neurons. The total number of trainable parameters of this model was 630,319.

#### 2.4.2. Customized Convolutional Neural Network

**Figure 3:**
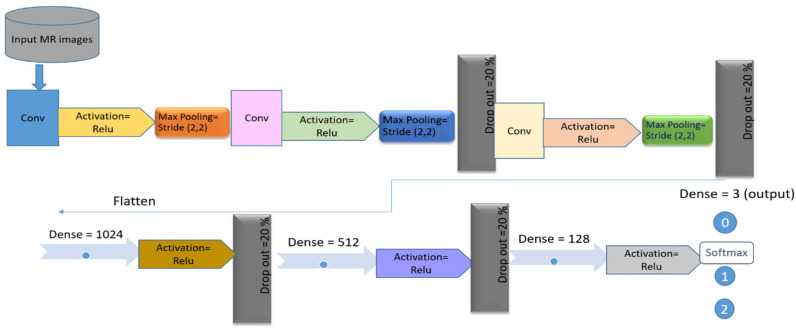
Customized convolutional neural network architecture with eleven layers for the prediction of anterior cruciate ligament tears.

The second variant was a more enhanced version of the standard CNN described in Section 3.2. Our customized CNN model used a total of 11 layers excluding input and output layers. These include three layers of a combination of convolutional maximum pooling (Conv-pool) and ReLu activation. We used the same parameter settings as in our standard CNN model with 20 filters, a kernel size of 5 × 5, a stride of 2 × 2, and max-pooling (2 × 2) to learn more features. Three dense layers (fully connected layers) were added after Conv-pool layers with 1024, 512, and 128 neurons, respectively. Four dropout layers were also included for regularization and to avoid over-fitting of the model after the second and third Conv-pool layer and after the first and second dense layer. We used the softmax activation function to get the probabilities of all three classes. The customized CNN model contains 3,090,515 trainable parameters in the last layer. Figure 3 illustrates the customized CNN model with four dropout layers.

### 2.5. Proposed Work Framework

**Figure 4:**
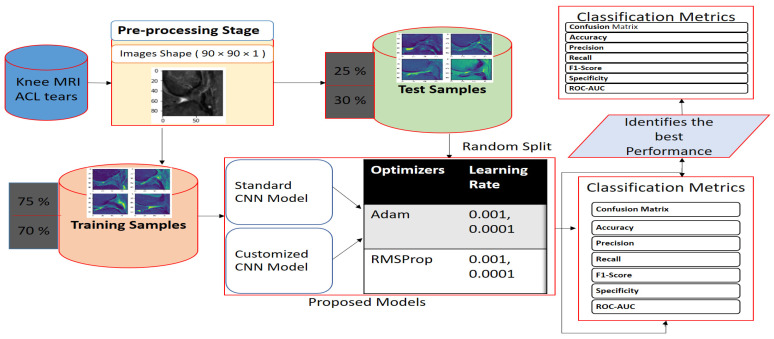
Graphical representation of the block architecture of our framework.

The overall framework of our deep learning approach to detect ACL tears consisted of three steps: (1) a pre-processing stage, where knee MR image slices with midmost measurements (320 × 320 × 32) were cropped to the region of interest at a fixed dimension of (90 × 90 × 1); (2) a hyper-parameter adjustment stage, where we trained the proposed standard CNN and a customized CNN model as described in Section 3.2. We manually set the optimizer’s adaptive moment estimation (Adam) [51], the optimizer’s root mean square propagation (RMSprop) [52], and two learning rates of 0.001 and 0.0001, which trained well; and (3) identification of the best performance on different evaluation metrics with a random split into training (75%, 70%) and test samples (25% and 30%). The graphical representation of the block architecture of our framework is shown in Figure 4.

## 3. Experimental Results

This section presents the experimental framework and hyper-parameters to analyze our models and evaluate the results.

### 3.1. Implementation Details

The experiments were performed out on accelerated Google Colab [53] cloud service, which provides CPU of Intel(R) Xeon(R) CPU @ 2.20GHz, GPU of Nvidia-Tesla T4, and 12 GB of RAM. Python 3.7 was used along with Numpy, Pandas, Scikit-learn, Tensorflow 2.5.0, and Keras 1.5.

### 3.2. Train and Test Random Splitting

**Figure 5:**
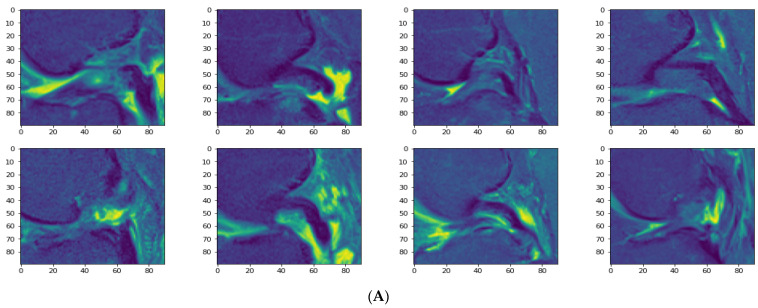

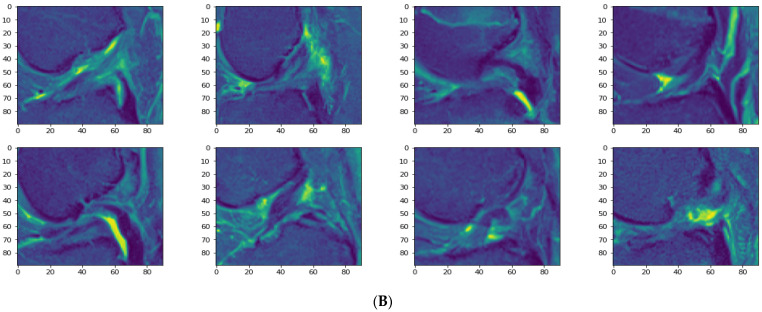
Samples of training and test datasets; (**A**) visualization of eight training sample images; (**B**) eight test sample images.

Our models were trained and tested after dividing the dataset by applying a random split. For each approach, we divided our data set with a test split ratio of 25% and 30%, respectively. The were 827 training samples with a 75% training split and 276 test samples representing a ratio of 25%. In the case of a 70% training split, the number of samples was 772, and 331 samples were held out for the test dataset at a 30% ratio. The visualization of 8 images is shown in Figure 5A of the training and test datasets in Figure 5B.

### 3.3. Hyperparameter Adjustments of our Models

Parameters are the weights and biases, whereas hyper-parameters are variables that determine a convolutional network’s structure, such as the number of neurons, hidden layers, learning rate, the number of epochs, optimizer, batch size, and activation functions to manually make the CNN more efficient. The hyper-parameter adjustment of our models regarding learning rate value, optimizer technique which we adapted, the number of epochs, batch size, and the number of layers employed was determined by the CNN architecture. Our standard CNN and customized CNN models used two fast optimizers, RMSprop and (Adam), to get good results.

- The RMSProp optimizer tries to dimple the auscultations. It fixes the convergence problem to global minima in the adaptive gradient (AdaGrad) optimizer by accumulating only the gradients from the recent iterations. RMSprop chooses different learning rates for each parameter. RMSprop updates as mentioned in Equation (5). The value of the beta decay rate is close to 0.0001. The weights are updated as shown in Equation (6).

(5) $$v_{t=Bv_{t-1}+\left(1-B\right)\;*\;}g_{t}^{2}$$

(6) $$w_{new}=w_{Old}-\frac{n}{\sqrt{v_{t^{+\varepsilon }}}}*\;g_{t}$$

- Adam is a well-known optimizer with good performance when it comes to classifying images in CNNs. It is a variant of a combination of RMSprop and momentum. It uses an estimation of the first and second momentum of gradients to adapt the learning rate for each weight of the neural network. Adam also makes use of the average of the second moments of the gradients. The algorithm calculates an exponential moving average of the gradient and the squared gradient, and the parameters beta1 and beta2 control the decay rates of these moving averages in Equations (7)–(9).

(7) $$\theta_{t+1}=\theta_{t}-\frac{n}{\sqrt{v_{t}}+\varepsilon }m_{t}$$

(8) $$m_{t=}\beta_{1}m_{t-1}+\left(1-B_{1}\right)g_{t}\;where\;m_{t=\frac{m_{t}}{1-B_{1}^{t}}}$$

(9) $$v_{t=}B_{2}v_{t-1}+\left(1-\beta_{2}\right)g_{t}^{2}\;where\;v_{t}=\frac{v_{t}}{1-B_{2}^{t}}$$

### 3.4. Evaluation Metrics

The performance of the proposed techniques, we used a confusion matrix, precision (also known as positive predicted value), accuracy, recall (also known as hit rate, sensitivity, or true positive rate (TPR)), selectivity (also known as specificity or true negative rate (TNR)), F1 score, categorical cross-entropy, receiver operating characteristics (ROC) curve, and area under the curve (AUC). The evaluations metrics are described below.

1. Confusion matrix

A confusion matrix is based on an M × M matrix where M is the predicted number of classes. In our case, we had three classes; hence, our confusion matrix was 3 × 3. The confusion matrix has four outcomes: true positives (TP), those belonging to the class and correctly classified in that class; true negatives (TN), those not belonging to the class and correctly classified in another class; false positives (FP), also called type-I error, those not belonging to the class and wrongly assigned to the class; and finally, false negatives (FN), also called type-II error, those belonging to the class and mistakenly classified in another class.

2. Accuracy

The average accuracy of the model is calculated as the fraction of the total samples correctly classified, that is truly positives and true negatives. Accuracy is calculated as in Equation (10) below.

(10) $$Accuacy=\frac{Number\;of\;True\;Postive+Number\;of\;True\;Negative}{Total\;Number\;of\;healthy,\;partial\;and\;full\;ruptutured\;tears}$$

3. Precision (or positive predicted value)

The fraction of correct positive images divided by the total number of true positives and false positives. Precision can be expressed as in Equation (11).

(11) $$Precison=\frac{Number\;of\;\mathrm{correctly}\;positively\;classfied\;images\;\left(TP\right)}{\;Number\;of\;TP+Number\;of\;FP}$$

4. Recall (or sensitivity, hit rate, or true positive rate)

The fraction of all positive images in three classes correctly predicted as positive by the classifier. The recall formula can be expressed as below in Equation (12).

(12) $$Recall=\frac{Number\;of\;\mathrm{correctly}\;positively\;classfied\;images\;\left(TP\right)}{\;Number\;of\;TP+Number\;of\;FN}$$

5. Specificity or true negative rate

The fraction of all negative images in the three classes correctly predicted as negative by the classifier. The specificity formula can be expressed as below in Equation (13).

(13) $$Specificity=\frac{Number\;of\;\mathrm{correctly}\;negatively\;classfied\;images\;\left(TN\right)}{\;Number\;of\;TN+Number\;of\;FP}$$

6. F1 score

It combines precision and recall through harmonic means. The formula of F1 score is given in Equation (14).

(14) $$F1\;Score=2*\;\frac{Precision*recall}{precsion+recall}$$

7. Categorical cross-entropy

It is a loss between multiple (more than two) classes. It is a softmax activation plus cross-entropy. If M samples belong to N classes, then categorical cross-entropy is calculated as in Equation (15). The negative sign is tedious to carry around. It is useful to simply maximize the log-likelihood.

(15) $$Categorical\;Crossentropy\;Loss=-\overset{N}{\underset{i=1}{\sum }}\overset{M}{\underset{j=1}{\sum }}t_{ij}\log y_{ij}$$

8. ROC AUC

ROC AUC indicates if the probabilities of the positive classes are separated from the negative classes in a good manner. In ROC, the x-axis represents the false-positive rate or 1-specificity, and the y-axis represents the true positive rate or sensitivity. We can use various threshold values to plot our sensitivity (TPR) and (1-specificity) FPR on the curve. Both values range between 0 and 1.

#### 3.4.1. Experimental Prediction Performance of Standard CNN Model

We compiled the standard CNN model with a softmax activation function, categorical cross-entropy loss function, Adam optimizer, RMSprop, a learning rate of 0.001 and a learning rate of 0.0001. We only trained the model with a batch size of 6, but we used 100 and 200 epochs. However, in the case of 100 epochs, our standard model did not perform with a good accuracy. We calculated the average accuracy, precision, sensitivity, specificity, F1-score, and AUC. Table 1 is our standard CNN model with optimizers Adam or RMSprop and a learning rate of 0.001 or 0.0001. The investigated ratios of test splits are 25% and 30%. The technique using the Adam optimizer with a learning rate of 0.0001 after 25% test split yielded excellent results in terms of accuracy, precision, sensitivity, specificity, and F1-score. However, in the case of AUC, the Adam optimizer with a learning rate of 0.001 after 25% and 30% test splits achieved the highest value of all techniques, 0.970, for the standard CNN.

#### 3.4.2. Experimental Prediction Performance of the Customized CNN Model

As the standard CNN model did not perform well in all test loss values of all approaches, there was a need to evaluate our customized CNN model. We predicted our modified CNN model again through average accuracy, precision, sensitivity, specificity, F1-score, and AUC. Table 2 is our customized CNN model with optimizers Adam or RMSprop and a learning rate of 0.001 or 0.0001. In the customized CNN model, the approach of RMS optimizer with a learning rate of 0.001 after 25% test split achieved an accuracy, precision, sensitivity, specificity, and F1-score of 98%. However, the Adam optimizer with a learning rate of 0.001 after 25% test split achieved an AUC of 0.990, the highest among all techniques in the case of the standard CNN.

#### 3.4.3. Result Comparison between Standard and Customized CNN Approaches

Here, we compared the results of our best performing standard CNN with the customized CNN in three classes of ACL tears by MRI in terms of confusion matrices, training and test model accuracy plots, and ROC AUC curves.

**Figure 6:**
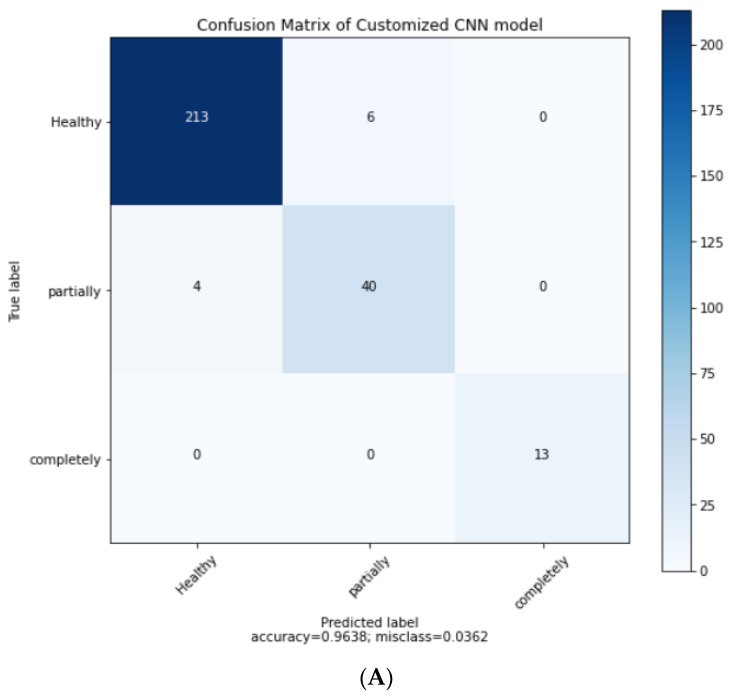

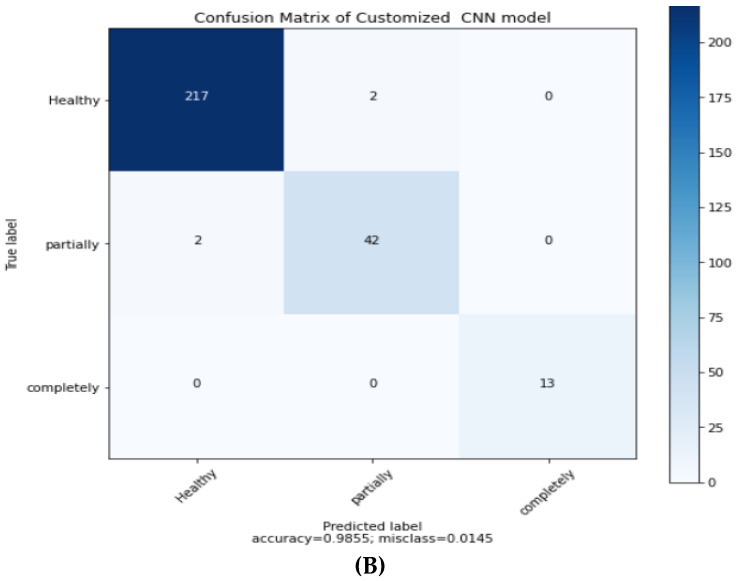
Confusion matrices. (**A**) The confusion matrix of the standard CNN model with Adam optimizer and a learning rate of 0.0001 after a 25% test split. (**B**) The confusion matrix of the customized CNN model with RMSprop optimizer and a learning rate of 0.001 after 25% test split.

Firstly, Figure 6 shows the value of healthy, partial, and fully ruptured tears in the confusion matrix. Then, the confusion matrix plots are taken from the best technique of both of our models. Figure 6A shows the confusion matrix of the standard CNN model with Adam optimizer and a learning rate of 0.0001. Similarly, Figure 6B shows the confusion matrix of the customized CNN model with RMS optimizer and a learning rate of 0.001 after a 25% test split.

**Figure 7:**
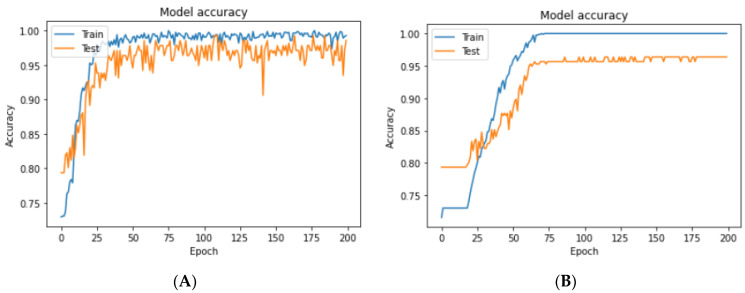
The accuracy plots of training and test datasets. (**A**) Training and test accuracy of the customized CNN with RMSprop optimizer and a learning rate of 0.001. (**B**) Training and test accuracy of the standard CNN with Adam optimizer and a learning rate of 0.0001.

Secondly, we plotted the accuracy of the training and test results of our models. A higher accuracy was achieved with the customized CNN after adjusting the hyper-parameters using three hidden layers of convolutional pooling, four dropout layers, the RMSprop optimizer, a learning rate of 0.001, and random splitting of 25%. In Figure 7A, the training and test accuracies of this model were compared in a plot where the test dataset accuracy was 98.6%. Figure 7B shows the test accuracy plot of the standard CNN model with an Adam-optimized learning rate of 0.0001 on a 25% split test set. An accuracy of 96.3% was achieved for the standard model. Thus, our customized CNN model performed with higher accuracy, precision, recall, specificity, F1-Score, and lower test loss values in some cases.

**Figure 8:**
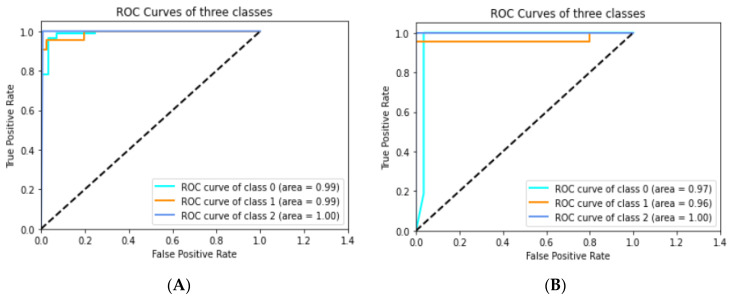
The ROC curve of three classes after 25% test split. (**A**) Customized CNN with Adam optimizer and a learning rate of 0.001. (**B**) Customized CNN with RMSprop optimizer and a learning rate of 0.001.

Thirdly, we plotted the ROC area under the curve of healthy, partially ruptured, and completely ruptured ACLs through customized CNN model approaches. For example, Figure 8A shows the values for the three classes (0.99, 0.99, and 1.00, respectively) of the Adam optimizer with a learning rate of 0.001 after a 25% test split; the average AUC was 0.990. In Figure 8B, the average AUC achieved was 0.976 in the case of the RMSprop optimizer with a learning rate of 0.001 after a 25% test split.

**Figure 9:**
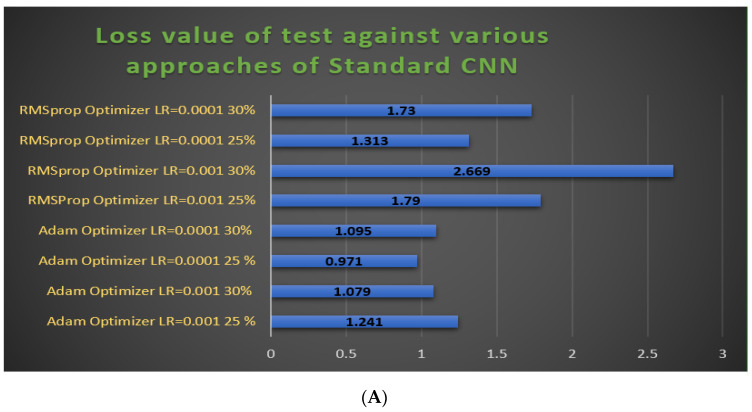

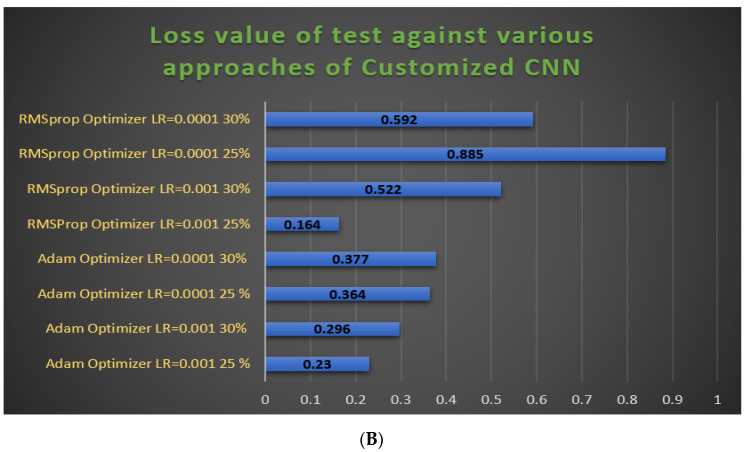
Bar graphs of loss value comparisons of our models. (**A**) The bar graph of the test loss values of eight approaches of the standard CNN model. **(B**) The bar graph of the test loss values of eight approaches of the standard CNN model.

In Figure 9A, the graph of the test losses of all approaches of the standard CNN was plotted. The value for the RMSprop optimizer with a 30% test split and a learning rate of 0.0001 is 2.669, which is the worst loss of all models. The minimum value was 0.971 for the Adam optimizer, a learning rate of 0.0001 after 25% test split. A lower loss value means the model error is smaller. In Figure 9B, a graph of the test loss values of all approaches of the customized CNN model is plotted. The value for the RMSprop optimizer with 25% test split and a learning rate of 0.0001 is 0.885, which is the worst of all models. The lowest test loss value is 0.164, achieved with the RMSprop optimizer, a learning rate of 0.001 with a 25% test split.

**Figure 10:**
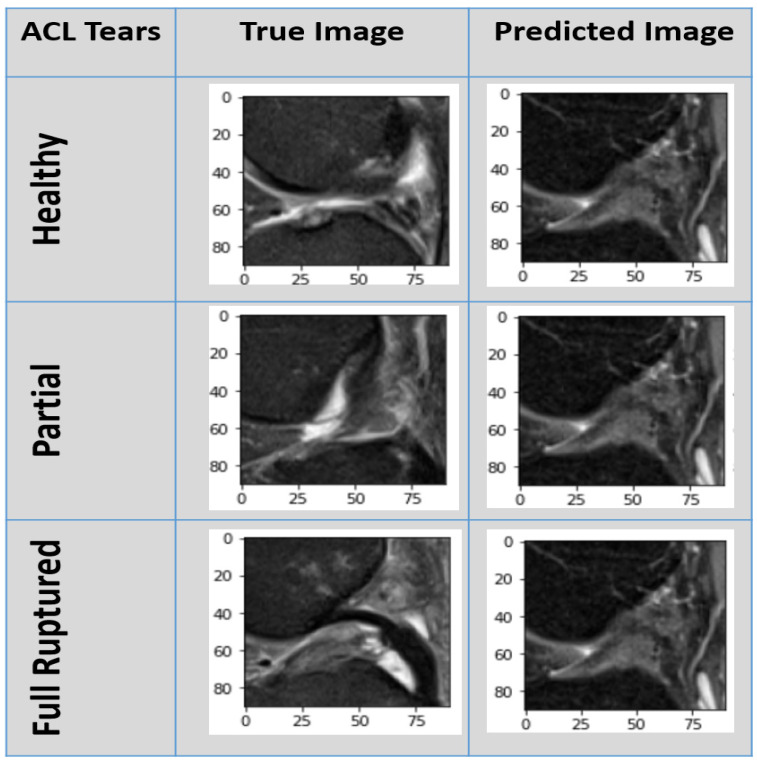
Our model predicted results against true images of healthy, partially ruptured, and fully ruptured ACLs.

Figure 10 displays the result of our customized CNN model against the healthy, partially ruptured, and fully ruptured ACL true images with corrected predicted images. As our model performance was above 96%, there was only a 4% chance of wrong prediction.

## 4. Discussion

The severe knee osteoarthritis stage is painful for those who suffer it. ACL tear is a common injury that accelerates joint degeneration and causes an osteoarthritis (OA) risk. Hence, there is a need to prevent ACL injury and reduce OA automatically and accurately in less time. This work aimed to identify and classify knee ACL tears from MR images and compare the performance of various evaluation metrics without human interpretation. Our results obtained through deep learning exhibited an excellent performance of the models that can classify ACL tears and prevent OA.

Previously, authors have used deep learning methodology to detect knee MRI ACL tears’ severity in two or three classes. To our knowledge, six studies described the performance on the same 917 ACL MRI tears and two studies on different ACL tear datasets but the same CNN model with a different approach.

The dataset of ACL tear MRIs was taken from Stajduhar et al.’s [41] study. Only AUC was measured in partial tears and fully ruptured tears, showing 0.894 and 0.943, respectively, after 10-fold cross-validation through linear support vector machines. Bien et al. [42] performed their study on partial tears with logistic regression on the validation dataset of knee ACL MRIs. The AUC of this study was 0.911. Tsai et al. [43] again performed their tests on the external dataset of ACL MRIs after 2-fold cross-validation with an AUC of only 0.913. Namiri et al. [45] achieved 94.6% specificity and a lower value of sensitivity (59.6%) with a 70:20:10 ratio of 1243 knee MRIs. The 3D CNN model also showed poor sensitivity with an average value of 63.3% of the three classes. Recently, Li et al. [54] only considered 60 ACLs, and the performance of the CNN model after applying feature fusion was 92.1% accuracy. Dunnhofer et al. [55] proposed the MRPyrNet architecture on ELNet and MRNet validated with a 20% split on the ACL MRI dataset. The accuracy, AUC, specificity, and sensitivity were 85%, 0.900, 90.8%, and 67.8%, respectively. Kapoor et al.’s model [46] performed with 88.8% accuracy on the ACL MRI dataset. State-of-the-art work by M. J. Awan et al. [47] used the ResNet-14 model after a hybrid balancing of ACL MRI tears with a 25% random split and 5-fold cross-validation. The accuracy, AUC, precision, specificity, sensitivity, and test loss were 92%, 0.980, 91.7%, 94.6%, 91.7%, and 0.466, respectively. We compared our proposed models, results, dataset, and criteria with eight previous studies in Table 3.

Previously, authors also used deep learning methodology to detect knee ACL tears on MR images but mostly identified only two classes. Furthermore, the approaches were time consuming in the case of radiologist involvement and did not achieve good accuracies and AUCs.

Our study has several limitations. First, the imbalanced dataset; the share of healthy images is much higher than those of partially and fully ruptured tear images. Second, the patients’ information is not available regarding their ages, demographic location, and history of ACL injury. The study not considered patients with ACL rupture for young and a history of trauma or osteoarthritis. Third, the study is not evaluated through cross-validation. In the future, we can validate our model on other datasets as an external validation and check the results after class balancing.

## 5. Conclusions

We developed a deep learning model that achieved the highest performance for prospective classification and demonstrated the benefit for patients with osteoarthritis. We present state-of-the-art work based on a customized CNN model after the adjustment of hyper-parameters. The proposed CNN model has multiple hidden layers, dropout layers, the RMSprop optimizer, a learning rate of 0.001 and achieved an accuracy, precision, specificity, and sensitivity above 98%. The results revealed that the deep learning-based CNN model substantially improved the classification of knee ACL tears, also in terms of AUC. To the best of the authors’ knowledge, there is no such study with an accuracy, precision, specificity, sensitivity, and area under curves of above 98%. Our proposed model had a test loss of only 0.164. The AUC value was 0.990 in the case of the Adam optimizer with a learning rate of 0.001. This model can be applied to other knee ligament injuries.

## Figures and Tables

**Table 1:** The evaluation metrics of three classes average with our standard CNN model.

Model	Evaluation Metrics					
Standard CNN Techniques	Accuracy	Precision	Sensitivity	Specificity	F1-Score	AUC
Adam Optimizer LR = 0.001 25%	94.2%	91.6%	95.3%	95.9%	93.0%	0.970
Adam Optimizer LR = 0.001 30%	93.3%	88.3%	90.0%	95.4%	89.0%	0.970
Adam Optimizer LR = 0.0001 25%	96.3%	95.0%	96.0%	96.9%	95.6%	0.950
Adam Optimizer LR = 0.0001 30%	93.3%	88.3%	90.0%	96.9%	89.0%	0.960
RMSprop Optimizer LR = 0.001 25%	94.2%	89.3%	95.3%	95.4%	92.3%	0.966
RMSprop Optimizer LR = 0.001 30%	92.1%	83.6%	88.0%	94.4%	85.6%	0.950
RMSprop Optimizer LR = 0.0001 25%	94.2%	85.6%	93.6%	95.9%	89.3%	0.956
RMSprop Optimizer LR = 0.0001 30%	92.7%	87.6%	89.6%	95.2%	88.6%	0.950

**Table 2:** The evaluation metrics of three classes averaged with our customized CNN model.

Model	Evaluation Metrics					
Customized CNN Techniques	Accuracy	Precision	Sensitivity	Specificity	F1-Score	AUC
Adam Optimizer LR = 0.001 25%	97.1%	96.3%	96.3%	97.0%	96.3%	0.990
Adam Optimizer LR = 0.001 30%	97.0%	97.0%	92.6%	96.9%	94.3%	0.983
Adam Optimizer LR = 0.0001 25%	96.3%	95.0%	96.0%	96.9%	95.6%	0.970
Adam Optimizer LR = 0.0001 30%	95.1%	92.0%	93.6%	95.5%	92.6%	0.976
RMSprop Optimizer LR = 0.001 25%	98.6%	98.0%	98.0%	98.5%	98.0%	0.976
RMSprop Optimizer LR = 0.001 30%	94.0%	90.6%	87.6%	93.8%	89.3%	0.953
RMSprop Optimizer LR = 0.0001 25%	94.6%	92.0%	95.3%	96.0%	93.6%	0.976
RMSprop Optimizer LR = 0.0001 30%	91.8%	87.3%	91.3%	93.6%	89.3%	0.966

**Table 3:** Comparison of state-of-the-art works with our proposed model.

Studies	Train/Test/Validation Split % & Dataset	Target ACL Tears	Experimental Techniques	Evaluation					
				Accuracy	AUC	Precision	Specificity	Sensitivity	Test Loss
Stajduhar et al., 2017 [41]	10-fold cross-validation 917 ACL MRI cases	Partially	HOG + Lin SVM	-	0.894	-	-	-	-
		Fully ruptured	HOG + RF	-	0.943	-	-	-	-
Bien et al., 2018 [42]	60:20:20 Knee MRI validation: 183 ACL MRI	Partial, ruptured	Logistic Regression	-	0.911	-	-	-	-
Tsai et al., 2020 [43]	5-fold ACL:129	Ruptured	ELNet (K = 2) MultiSlice Norm + Blurpool	-	0.913	-	-	-	-
Namiri et al., 2020 [45]	70:20:10 1243 Knee MRI NIH	Average 3 classes ACL	2D CNN	-	-	-	94.6%	59.6%	-
		Average 3 classes ACL	3D CNN	-	-	-	93.3%	63.3 %	-
Dunnhofer et al., 2021 [55]	5-fold 80:20 917 ACL MRI	Average 3 classes ACL	MRNet with MRPyrNet	83.4%	0.914	-	84.3%	80.6%	-
			ELNet with MRPyrNet	85.1%	0.900	-	90.8%	67.9%	-
Kapoor et al., 2021 [46]	917 ACL MRI	Average 3 classes ACL	CNN	82.0%	-	-	-	-	0.42
			DNN	82.0%	-	-	-	-	0.43
			RNN	81.8%	-	-	-	-	0.45
			SVM	88.2%	0.910	-	-	-	
M. J. Awan et al., 2021 [47]	75:25 917 ACL cases	Average 3 classes ACL	Customized ResNet-14 + Class balancing Adam, LR: 0.001	90.0%	0.973	89.0%	94.0%	88.7%	0.526
	5-fold 917 ACL cases			92%	0.980	91.7%	94.6%	91.7%	0.466
Li et al., 2021, [54]	MRI group + Arthroscopy group ACL:60 cases	Grade 0 Grade 1 Grade II Grade III	Multi-modal feature fusion Deep CNN	92.1%	0.963	-	90.6 %	96.7%	-
**Proposed**	70:30 75:25 917 ACL MRI	Average 3 classes ACL	Standard CNN Adam LR = 0.0001, 25%	**96.3%**	**0.950**	**95.0%**	**96.9%**	**96.0 %**	**0.971**
**Proposed**	70:30 75:25 917 ACL MRI	Average 3 classes ACL	Customized CNN Adam, LR = 0.001, 25%	**97.1%**	**0.990**	**96.3%**	**97%**	**96.3%**	**0.230**
			Customized CNN RMSprop, LR = 0.001, 25%	**98.6%**	**0.976**	**98.0%**	**98.5%**	**98.0%**	**0.164**

The bold parts are author’s approaches.

## Data Availability

We are using this dataset kneeMRI dataset available online: http://www.riteh.uniri.hr/~istajduh/projects/KneeMRI/ (accessed on: 1 March 2017) in our work from Clinical Hospital Centre Rijeka, under reference [41].
